# Node dissection in prostate cancer: no answers for old questions

**DOI:** 10.1590/S1677-5538.IBJU.2021.1063.1

**Published:** 2021-12-21

**Authors:** Rodolfo Borges dos Reis, Antônio Antunes Rodrigues, Rafael Neuppmann Feres, Marcelo Cartapatti da Silva, Valdair Francisco Muglia

**Affiliations:** 1 Universidade de São Paulo Faculdade de Medicina de Ribeirão Preto Departamento de Cirurgia e Anatomia Ribeirão Preto SP Brasil Departamento de Cirurgia e Anatomia, Faculdade de Medicina de Ribeirão Preto, Universidade de São Paulo, Ribeirão Preto, SP, Brasil; 2 Universidade de São Paulo Faculdade de Medicina de Ribeirão Preto Departamento de Imagens Médicas, Radioterapia e Oncohematologia Ribeirão Preto SP Brasil Departamento de Imagens Médicas, Radioterapia e Oncohematologia, Faculdade de Medicina de Ribeirão Preto, Universidade de São Paulo, Ribeirão Preto, SP, Brasil

## COMMENT

Over the past century, it has been recognized that pelvic and abdominal lymph nodes are common sites for metastatic prostate cancer (1). Flocks (2) reported that metastatic nodes were also frequently found in early prostatic cancer, this finding, associated with the development of safe surgical techniques to remove the prostate gland, has brought attention to the importance of pelvic lymphadenectomy. The presence of positive lymph nodes is associated with a worse prognosis and every effort should be made to detect the lymph node involvement and establish the best therapeutic plan. Important questions have arisen since then: how extensive should the pelvic lymph node dissection (PLND) be? is PLND a staging or therapeutic procedure?

### Evidences from pre-PSA era:

The template for PLND suggested by Whitmore and Mackenzie (3) included the external iliac nodes, the hypogastric nodes, and the obturator region, it was called the standard lymph node dissection. McCullough et al. included the common iliac nodes and even the para-aortic nodes (4). Paulson et al. suggested that a limited dissection, compromising just the hypogastric and obturator nodes, would be as efficient as the standard, reducing operative time and complications (5). Flocks reported a 13% tumor-free survival in patients who had positive nodes, with radioactive gold implantation at the time of surgery (6). Barzell et al. initially suggested that tumor and lymph nodes volume could be a predictor to remain disease-free at 5 years (7). Prout et al. showed that only 18% of patients with solitary lymph node metastases developed metastatic disease (8). Based on these findings, Golimbu et al. suggested to extend the template dissection, to the presacral and presciatic nodes (9). Later Grossman et al. reported that patients with only one positive node did no better than those with multiple nodal metastases in terms of developing metastatic disease, although a longer disease- free survival was correlated with the number of nodes involved (10).

### Evidence in the PSA-era:

The United States Food and Drug Administration (FDA) approved in 1986 the use of PSA test to monitor the progression of prostate cancer (11), for this specific indication PSA is a good biomarker and revolutionized the way we follow patients and created a new definition of disease relapse. Since then, the term biochemical recurrence (BR) was introduced.

In 2008 Mattei et al. proposed to extend the dissection areas. The new template included the external and obturator regions, the portions medial and lateral to the internal iliac vessels, and the common iliac artery, at least up to the ureteric crossing (12). The authors stated that by removing the nodes from these regions approximately 75% of all nodes potentially harboring metastasis would be removed.

Today, two main questions are still on debate: how extensive should the pelvic lymph node dissection (PLND) be? is PLND a staging or therapeutic procedure?

Guidelines recommend extended PLND (ePLND) for patients with localized disease based on the risk of lymph node involvement (LNI). The European Urological Association adopted a cut-off risk > 7% attested by the updated Briganti's nomogram (13), while the American Urological Association set the cut-off risk at 2% (14, 15). Despite the recommendations for ePLND, there was no randomized clinical trial comparing PLND yes vs no, or ePLND vs limited, addressing the oncological benefit of this approach.

For comparative assessments, the National Institutes of Health (NIH) and the FDA emphasize the importance of randomization. Today the impact of PLND on overall survival and quality of life are based on retrospective studies. Although some, particularly those enthusiastic about big data, would argue that the “real-world” retrospective observational studies can generate enough information to help the decision-making process, others will argue against it, emphasizing that at most these studies can be hypothesis generators. The debate is open, but until now there is no data to support the PLND oncological benefit. Overall survival can be affected by aggressiveness, staging, access to subsequent therapies, and global health. As a rule, the published retrospective series available are very heterogeneous, with a lack of information, even regarding the node dissection extension.

The role of PLND in avoiding biochemical recurrence (BCR) was addressed by two prospective randomized trials comparing ePLND x more limited dissection (16, 17). Overall, both trials found no difference in BCR. The study by Lesting et al. is criticized for including patients with biopsy International Society of Urological Pathology (ISUP) grade 1 or 2, 79% of the sample, and a median prostate-specific antigen of 10.5 ng/mL, a population unlikely to recur in 5 years.

The study by Touijer et al. is also criticized, the number of nodes resected in both groups was very similar, 12 in the limited dissection arm, vs 14 in the extended arm, suggesting that the resection template was not appropriately followed, making comparisons difficult. However, they are the best evidence available, and both are pointing in the same direction.

Despite PLND being the best method for lymph node staging and influencing subsequent treatments, and the incredible number of emerging therapies for PCa, today there is no unquestionable proof that PLND can improve overall survival, the ultimate endpoint of interest for cancer patients.

Taking together, the questions from the pre and PSA era are still under debate. Further studies with longer follow-up are necessary to have the right answers. I congratulate the authors for their work in selecting the best evidence available and presenting it clearly and concisely (18).
